# Unexplained Portal Gas in a Patient with an Esophageal Ulcer

**DOI:** 10.1155/2018/2496193

**Published:** 2018-12-10

**Authors:** Samiksha Gupta, Balaram Krishna Surapaneni, Rakesh Vinayek, Sudhir K. Dutta

**Affiliations:** ^1^Sinai Hospital, Baltimore, Maryland, USA; ^2^Aventura Hospital and Medical Center, Aventura, Florida, USA

## Abstract

Emphysematous gastritis is the infection of gastric mucosa by gas producing microorganisms. It is a rare infection with less than 100 cases reported in the literature. The association of portal venous gas along with emphysematous gastritis is a rare entity. The concomitant portal venous gas worsens the outcome and warrant for surgical treatment. Our case has portal venous gas on CT scan along with suspicion of emphysematous gastritis and an esophageal ulcer on upper GI endoscopy. Medical treatment was given in our case of portal venous gas with the esophageal ulcer. Our case is unique because our patient responded to the conservative management. The patient presented with past history of polysubstance abuse and chronic kidney disease presented with symptoms of acute abdomen. CT scan revealed portal venous gas and suspicion of gastric emphysema. In addition, few foci of gas are seen along the vessels traversing between the stomach and liver. Endoscopy with gastric mucosa biopsy showed Candida albicans. Subsequently, antifungals were started. There was improvement in clinical condition of the patient. We, hereby, also summarize all the reported cases of emphysematous gastritis with treatment and outcome in each case. There has been change in trend from surgical to medical treatment.

## 1. Introduction

Emphysematous gastritis (EG) is a polymicrobial infection of gastric mucosa that is caused predominantly by gas producing bacteria. Though EG is a rare condition with a handful of cases reported, it is associated with a poor prognosis. In addition, the presence of portal venous gas in individuals with EG further worsens the outcome. Until recent years, surgery has been the main modality of treatment for emphysematous gastritis, especially in patients with associated portal venous gas. However, there has been a change in trend from the surgical management to a more conservative approach because of the postsurgical complications and insignificant difference in long-term prognosis between two approaches.

Herein, we report a case of a middle aged male with past medical history of polysubstance abuse and chronic kidney disease who was suspected of emphysematous gastritis with associated portal venous gas. Our case report is unique because our patient responded adequately to the conservative treatment despite the associated portal venous gas.

## 2. Patient Information

A 48-year-old man with past medical history of COPD on home oxygen, chronic anemia, remote history of polysubstance abuse and alcohol abuse, tobacco use, and chronic kidney disease, PUD gastritis, and GI bleeding, presented to our hospital with complaints of fever, sudden onset nausea, abdominal pain, and hematemesis for 3 days.

## 3. Clinical Findings

On physical examination, his abdomen was distended and there was tenderness on palpation over the epigastrium, right and left upper quadrants but without any guarding and rebound tenderness. In addition, the bowel sounds were hypoactive. His vital signs were unremarkable apart from mild tachycardia.

## 4. Timeline

Acute onset of pain with upper GI bleeding was suggestive of borderline features of acute abdomen.

## 5. Diagnostic Assessment

**Figure 1:**
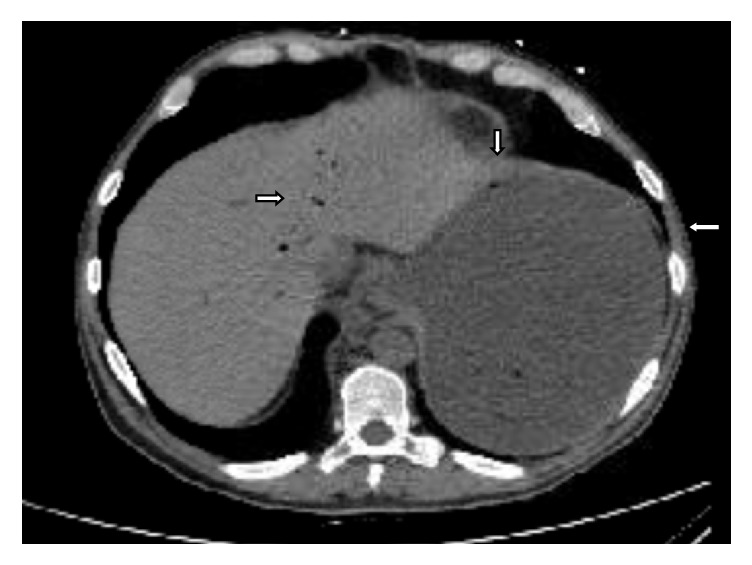
Abdominal CT scan showing stomach distension and limited gas within the wall of the stomach and in the portal venous system.

Our patient's CBC with hemoglobin of 7 mg/dl and hematocrit of 27.6% was diagnostic of anemia and leukocytosis with WBC count of 16.04 ×10^9^/L. His basic metabolic panel was significant for deranged kidney functions and electrolyte disturbances with elevated creatinine (1.97 mg/dl) and BUN (30 mg/dl), mild hypokalemia (3.2mEq/L), and hyperphosphatemia (8mg/dl). LFTs and lactate were within normal range. Furthermore, his CT scan revealed irregular thickened distal esophagus with markedly distended stomach (probably secondary to gastric outlet obstruction) as well as portal venous gas. There was suspicion of limited gastric emphysema as well. In addition, few foci of gas are seen along the vessels traversing between the stomach and liver (Figure 1).

## 6. Therapeutic Intervention

**Figure 2:**
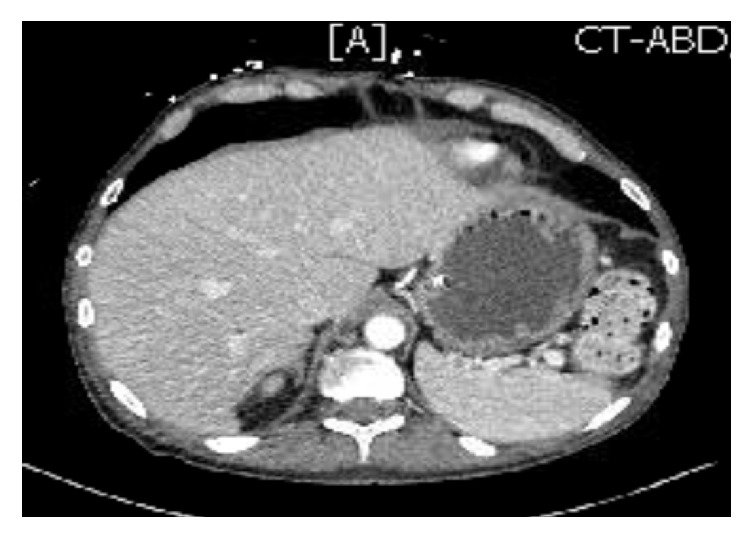
CT scan of the abdomen on the 5th day of admission showing resolution of air in the stomach wall and portal venous gas.

**Figure 3:**
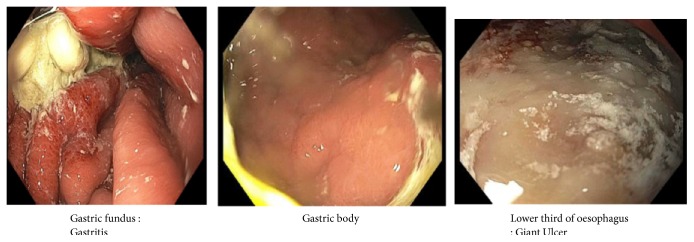
Endoscopy images showing nonbleeding erosive gastropathy, erythematous mucosa in the gastric body and antrum, esophagitis, and nonbleeding esophageal ulcer (7th day of admission).

In view of the suspicion of emphysematous gastritis, our patient was kept nil by mouth, had an NGT decompression, and was initiated on intravenous PPIs. Also, intravenous piperacillin-tazobactam was added for the broad spectrum coverage against gram-negative bacteria and anaerobes. For his severe anemia, he was given a unit of blood and his hemoglobin/hematocrit monitored 6 hourly. The patient had a repeat CT scan on the 5th day of admission which showed partial resolution of the gas on the CT scan. (Figure 2). On the 7th day of admission, he had an upper GI endoscopy which was suggestive for nonbleeding erosive gastropathy, erythematous mucosa in the gastric body and antrum, esophagitis, nonbleeding esophageal ulcer (Figure 3). However, there were no gross findings suggestive of ischemia or necrosis. During the endoscopy, a biopsy was taken from the fundus, body, antrum of the stomach and distal esophagus. The biopsy histology was evident for mild chronic gastritis and there was the growth of Candida albicans in the culture. Our patient was started on intravenous fluconazole to which he responded well with improvement in his symptoms.

## 7. Follow-Up and Outcomes

Patient was followed up after 6 months and was asymptomatic.

## 8. Discussion

Emphysematous gastritis is a not so common entity with less than hundred cases reported till now on pubmed search. Emphysematous gastritis occurs when the gas producing microorganisms invade the wall of stomach and ferment glucose to produce gases like carbon dioxide (CO_2_) and nitrogen (N_2_). Clostridium, Streptococci, Staphylococcus, E. coli and Pseudomonas species, Candida and mucor constitute the group of organisms responsible for infection in EG [1]. A recent case report also mentions about the association of a rare gram-positive coccus, Sarcina ventriculi [2]. Although the Candida was detected in gastric mucosa biopsy in our case, it is difficult to mention that it is the causal agent as Candida is commonly associated with other comorbidities like CKD and immunosuppression. Gas chromatography has been done to find the type of gases in pneumatosis intestinalis; however, it has not been done in cases of emphysematous gastritis. Levitt MD et al. found that the intraluminal gas in intestine is the swallowed air by demonstrating the intraluminal gas composition similar to that of atmospheric air with predominantly nitrogen and oxygen less than 2 % [3]. However, the intramural gas composition in upper GI tract is unknown although it is clear that methane, hydrogen, and hydrogen sulphide found in cases of pneumatosis intestinalis are produced by bacteria in the intestine. These gases are not found in the atmospheric air in the same composition.

Ingestion of corrosives, alcohol abuse, recent surgery, compromise of blood supply due to atherosclerosis and other comorbidities like end-stage renal disease, peptic ulcer disease, diabetes mellitus, malnutrition, NSAIDs, and steroid use are certain factors that increase the risk of infection in gastric mucosa. In addition, hydrogen peroxide [4], AIDS [5], coxsackie B3 myocarditis [6], breast cancer [7], severe vomiting [8], and blunt abdominal trauma [9] have also been reported to be predisposing factors for EG. Our patient had a history of polysubstance abuse, smoking, hyperlipidemia, history of chronic kidney disease, and chronic anemia, which could precipitate emphysematous gastritis.

Diagnosis of emphysematous gastritis can be made on the basis of clinical features and characteristic findings on imaging. Clinically, in addition to acute abdomen symptoms of pain, nausea, or vomiting, EG also presents with fever, neurologic changes, and hemodynamic instability [10, 11]. Nonetheless, there can be a less dramatic presentation of emphysematous gastritis in patients with immunocompromised status like those of diabetes mellitus, end-stage renal disease, and cirrhosis [12]. The pathognomic finding of EG is emesis of the necrotic mucosal clot in the shape of the gastric wall that is due to dissection of the muscularis mucosa by bacterial organisms [13]. Clinical findings are mostly nonspecific in case of emphysematous gastritis. Therefore, radiological imaging plays an important role in the diagnosis of EG. Plain film shows distended stomach with thickened gastric folds due to edema along with innumerable gas bubbles in mottled distribution. CT scan is more sensitive compared to the plain films and is the modality of choice for making the diagnosis of EG [14]. With increasing use of CT scan worldwide, there is increase in the incidence and the prevalence of this entity. On CT scan, EG is characterised by the presence of gas bubbles in irregular pattern along the wall of stomach, thickening of gastric mucosal folds with or without portal venous gas [15]. Concomitant small bowel or colonic pneumatosis found on CT scan worsens the prognostic outcome [16]. Our patient was hemodynamically stable with worse CT scan findings compared to his clinical presentation. The CT scan showed portal venous gas and suspicion of gas within the gastric wall. Entry of gas in venous system occurs due to necrosis of bowel from infection, inflammation or rise in the intraluminal pressure [17]. Thus, presence of portal venous gas in patients with EG signifies poor prognosis [18]. In a study by Liebman et al., portal vein gas is associated with 75% mortality and almost all patients require surgery [19]. Due to severe abdominal pain and hematemesis with bubbly irregular air in the gastric wall without any history of mechanical trauma to abdomen, our case was suggestive of emphysematous gastritis. Radiologically, this case is unique because our patient responded well to adequate treatment despite the presence of portal vein gas. Very few cases of gastric emphysema with portal venous gas have been reported in the literature which responded to the conservative management [17, 20].

Due to the rare occurrence of disease, there are no established guidelines for diagnosis and management of emphysematous gastritis. During the past decades, patients used to present in advanced stage of emphysematous gastritis with ischemic and necrotic changes of gastric mucosa, peritonitis, and hemodynamic instability [22]. In such cases, gastrectomy or partial gastrectomy was considered as the treatment modality. Surgical intervention can be considered depending on presentation, hemodynamic status, age, comorbidities, the extent of bowel involvement, complications such as perforation of bowel, peritonitis, and necrotic bowel. The surgical intervention is usually associated with poor and unsatisfactory outcome especially in the setting of associated comorbidities [42]. In addition, there can be postsurgical complications including anastomotic breakdown and leakage, development of fistulae, and chronic stricture formation. There is 60%-80% mortality rate of patients with emphysematous gastritis despite early aggressive treatment [21]. Endoscopy with biopsy and culture of gastric mucosa and supportive medical treatment is preferred over surgical treatment because of the higher mortality rate associated with surgical interventions. Therefore, surgical management can be considered only when the conservative management fails. Matsushima K et al. listed the treatment and the outcome of 39 case reports published from 1980-2012 [10]. Further, we have added 28 cases of EG from 2012 until today (Table 1). On reviewing the literature, it is revealed that the rate of reporting has increased from 39 cases in 32 years (1980-2012) to 26 cases in 5 years (2013-2018). It is certain that the rate of reporting has quadrupled after 2012. This could perhaps the result of increased application of CT scan early on during the patient evaluation and increased sensitivity of CT scan for detection of intramural gas with technological enhancement. Although the diagnosis is suspected based on clinical features which are nonspecific, the actual diagnosis is made by radiological findings on CT scan. Increased application of CT scan in making diagnosis not only increased the rate of reporting but has also resulted in increased survival of EG patients due to early diagnosis. 20 out of 39 patients died from in 32 years (1980-2012) whereas 4 out of 28 cases died in 5 years (2013-2018). The increased survival could also be the result of early intervention in the form of broad spectrum antibiotics.

Gas within gastric wall could be of two origins. It could be intraluminal air dissecting the wall of stomach that occurs in case of gastric emphysema. Secondly, gas could be produced by fermentation process of bacteria or fungi like that in case of emphysematous gastritis [44]. Gastric emphysema is another condition that should be differentiated from emphysematous gastritis. Gastric emphysema is a clinical entity that occurs when the intraluminal air invades the wall of stomach secondary to traumatic (endotracheal intubation/ nasogastric tube placement), mechanic (increase pressure in bowel lumen due to obstruction), or inflammatory (ingestion of corrosive substances, ischemia due to atherosclerosis) etiologies. In contrast to emphysematous gastritis, gastric emphysema has a benign course with no evidence of infection. Matsushima K et al. compared clinical presentation of 39 cases of emphysematous gastritis with that of 36 cases of gastric emphysema where they concluded that features of infection and systemic toxicity were associated with emphysematous gastritis only [10]. EG requires antimicrobials whereas gastric emphysema is treated conservatively with self-resolution. In our case, the gastric biopsy sample showed the growth of Candida albicans and he responded well to antifungals.

## Figures and Tables

**Table 1:** Treatment and outcome of cases of EG (1980 -2018).

**Year**	**Author**	**Surgery**	**Isolated organism**	**Outcome**
1982	Williford et al.	No	Strongyloides stercoralis	Dead
1986	de Lange et al.	No	Gram-positive cocci	Dead
1987	Bloodworth et al.	No	Klebsiella pneumoniae, Streptococcus	Dead
1989	Williamson et al.	Exploratory laparotomy	Candida spp.	Dead
1989	Monteferrante et al.	NA	Group D enterococci, E. coli	Dead
1990	Caruana et al.	No	Peritoneal fluid: Torulopsis glabrate, stomach biopsy: mixed gram negatives	Alive
1990	Moosvi et al.	Subtotal gastrectomy	Enterobacter aerogenes, Enterobacter, candida, pseudomonas	Alive
1992	Binmoeller et al.	No	Clostridium welchii	Dead
1994	McKelvie et al.	No	Gram-positive cocci, gram-positive rods	Dead
1995	Van Allan et al.	Total gastrectomy	Streptococcus viridans, yeast, fungi	Alive
1995	Lin et al.	Total gastrectomy	Klebsiella pneumonia, E. coli,	Alive
1996	Sud et al.	No	E. coli	Dead
1998	Bashour et al.	Exploratory laparotomy	Clostridium perfringens, yeast	Dead
1999	Cherney et al.	Total gastrectomy	Stomach pathology: mucormycosis, peritoneal fluid: Yersinia, Enterobacter	Dead
2001	Shipman et al.	No	Enterococcus spp	Alive
2002	Wong et al.	No	NA	Dead
2002	Van Mook et al.	No	Klebsiella pneumonia, Candida albicans	Dead
2002	Ho DC et al.	No	NA	Dead
2003	Yalamanchili et al.	Total gastrectomy	Candida glabrata, Candida krusei	Dead
2003	Gutierrez et al.	No	NA	Alive
2003	Buyl et al.	Exploratory laparotomy	Pseudomonas aeruginosa, Streptococcus	NA
2004	Ocepek et al.	Cholecystectomy	Clostridium difficile	Dead
2005	Allan et al.	No	None	Alive
2006	Abboud et al.	Exploratory laparotomy	NA	Dead
2006	Iqbal et al. [21]	No	NA	Alive
2007	Loi et al.	No	Pseudomonas spp, Acinetobacter spp	Alive
2007	Carlson et al.	No	None	Alive
2007	Lee et al.	No	Enterobacter cloacae	Dead
2007	Jung et al.	Total gastrectomy	Stomach pathology: Mucormycosis	Dead
2008	Al-Jundi et al. [13]	No	Gastric aspirate: Klebsiella pneumoniae	Alive
2009	Huang et al. [22]	Total gastrectomy	Blood/peritoneal fluid: E. coli	Dead
2009	Agarwal et al.	Total gastrectomy	NA	Dead
2010	Schorn et al.	No	None	Alive
2010	Dawwas et al.	No	Gastric mucosa: Candida glabrata	Alive
2010	Paul M et al.	No	Streptococcus viridans	Alive
2011	Arezzo et al.	No	NA	Alive
2011	Hassan et al.	No	None	Alive
2012	Ferrada	Wedge resection of stomach	NA	Alive
2012	Matsushima K et al. [10]	Exploratory laparotomy	None	Alive
2012	Ng A et al. [23]	No	NA	Alive
2012	X. Verhelst et al. [24]	Subtotal gastrectomy	Candida albicans	Alive
2013	Yu HH et al. [25]	Exploratory laprotomy	Klebsiella pneumoniae	Alive
2013	Wormer BA et al. [26]	No	NA	Alive
2013	Szuchmacher M et al [27]	Exploratory laparotomy	NA	Alive
2013	Ratuapli et al. [28]	No	Sarcina ventriculi	Alive
2013	Nair et al. [29]	No	NA	Alive
2014	Murchie et al. [30]	No	NA	Alive
2014	Yusef et al. [31]	No	None	Alive
2014	A Misro [32]	No	NA	Alive
2015	Alvarado-Lezama J et al. [33]	Total gastrectomy	Mucor	Alive
2015	F. Uysal et al. [34]	No	Gram-positive anaerobic spore-forming bacillus	Alive
2015	Takano et al. [35]	No	Escherichia coli and Enterococcus avium	Alive
2015	Matsunaga et al. [7]	No	NA	Dead
2015	Guillén-Morales C et al. [36]	No	NA	Dead
2016	Zamora Elson M et al. [37]	No	H. pylori	Alive
2016	Ashfaq et al. [6]	No	NA	Dead
2016	Jehangir A et al. [38]	No	NA	alive
2016	Sharma et al. [39]	No	NA	Alive
2016	Sarvari KP et al. [40]	No	Gram positive thick rods: clostridium	Dead
2016	Sulaimani M et al. [11]	No	NA	Unknown
2017	Nehme F et al. [41]	No	NA	Alive
2017	Yao J et al. [5]	No	None	Alive
2017	Li K et al. [4]	No	NA	Alive
2018	Alvin M et al. [2]	Exploratory laprotomy	Sarcina ventriculi	Alive
2018	Kyawzaw L et al. [42]	No	NA	Alive
2018	Gardenyes J et al. [43]	No	NA	Dead
2018	Our case	No	Candida albicans	Alive
